# Microbial Community Structure and Chemical Constituents in *Shinkiku*, a Fermented Crude Drug Used in Kampo Medicine

**DOI:** 10.3389/fnut.2020.00115

**Published:** 2020-07-31

**Authors:** Zitai Wang, Kayu Okutsu, Taiki Futagami, Yumiko Yoshizaki, Hisanori Tamaki, Takuro Maruyama, Kazufumi Toume, Katsuko Komatsu, Fumio Hashimoto, Kazunori Takamine

**Affiliations:** ^1^The United Graduate School of Agricultural Sciences, Kagoshima University, Kagoshima, Japan; ^2^Faculty of Agriculture, Education and Research Center for Fermentation Studies, Kagoshima University, Kagoshima, Japan; ^3^Division of Pharmacognosy, Phytochemistry and Narcotics, National Institute of Health Sciences, Kawasaki, Japan; ^4^Division of Pharmacognosy, Department of Medicinal Resources, Institute of Natural Medicine, University of Toyama, Toyama, Japan; ^5^Department of Horticultural Science, Faculty of Agriculture, Kagoshima University, Kagoshima, Japan

**Keywords:** *shinkiku*, *Hangebyakujutsutemmato*, *Aspergillus* sp., *Rhizopus* sp., ferulic acid

## Abstract

*Shinkiku* (Massa Medicata Fermentata) is a traditional crude drug used to treat anorexia and dyspepsia of elder patients in east Asia. *Shinkiku* is generally prepared by the microbial fermentation of wheat and herbs. *Shinkiku* is also used in Japanese Kampo medicine as a component of 半夏白朮天麻湯 (*Hangebyakujutsutemmato*). However, the quality of *shinkiku* varies by manufacture because there are no reference standards to control the quality of medicinal *shinkiku*. Thus, we aim to characterize the quality of various commercially available *shinkiku* by chemical and microbial analysis. We collected 13 *shinkiku* products manufactured in China and Korea and investigated the microbial structure and chemical constituents. Amplicon sequence analysis revealed that *Aspergillus* sp. was common microorganism in *shinkiku* products. Digestive enzymes (α-amylase, protease, and lipase), organic acids (ferulic acid, citric acid, lactic acid, and acetic acid), and 39 volatile compounds were commonly found in *shinkiku* products. Although there were some commonalities in *shinkiku* products, microbial and chemical characteristic considerably differed as per the manufacturer. *Aspergillus* sp. was predominant in Korean products, and Korean products showed higher enzyme activities than Chinese products. Meanwhile, *Bacillus* sp. was commonly detected in Chinese *shinkiku*, and ferulic acid was higher in Chinese products. Principal component analysis based on the GC-MS peak area of the volatiles also clearly distinguished *shinkiku* products manufactured in China from those in Korea. Chinese products contained higher amounts of benzaldehyde and anethole than Korean ones. Korean products were further separated into two groups: one with relatively higher linalool and terpinen-4-ol and another with higher hexanoic acid and 1-octen-3-ol. Thus, our study revealed the commonality and diversity of commercial *shinkiku* products, in which the commonalities can possibly be the reference standard for quality control of *shinkiku*, and the diversity suggested the importance of microbial management to stabilize the quality of *shinkiku*.

## Introduction

Massa Medicata Fermentata is a traditional crude drug used for treatment of anorexia and dyspepsia in elders in East Asia, called *shinkiku* in Japan, *shenqu* in China, and *singug* in Korea. *Shinkiku* is prepared from wheat (*Triticum sativum*), apricot kernel (*Prunus armeniaca*), red beans (*Phaseolus angularis*), polygonum (*Polygonum hydropiper*), sweet wormwood (*Artemisia apiacea*), and cocklebur (*Xanthium strumarium*). After mixing these materials, the mixture is fermented for a few days and subsequently dried to obtain *shinkiku* (1). Although *shinkiku* is manufactured in only China or Korea, it is also used in Japanese Kampo medicine as a component of “*Hangebyakujutsutemmato* (半夏白朮天麻湯),” which is used to treat dizziness and nausea (2, 3). Along with the increase in the consumption of *Hangebyakujutsutemmato*, the demand for *shinkiku* has also increased in recent years in Japan (4). However, the quality of *shinkiku* considerably differs with respect to the manufacture (5). This is because there are no reference standards for quality control of *shinkiku*. The voluntary standard in Japan stated that *shinkiku* should contain appreciable starch and reducing sugar, while that in Korea or China focused on the maximum ash or water content (1, 6, 7). The quality instability of *shinkiku* is a serious problem among the distributers of crude drugs in Japan. Thus, the characterization and standardization of commercial *shinkiku* are strongly needed to stabilize its medicinal grade quality.

A characteristic of *shinkiku* is the unique microbial fermentation process during its manufacture. “*Qu*” is sometimes mixed as a fermentative starter in China. *Qu* is the same as “*Koji*” in Japan, which is a solid-state culture of filamentous fungi (*Aspergillus* sp., *Rhizopus* sp., or *Mucor* sp.) on cereal grains, and is used to manufacture soy sauce, vinegar, and liquor. As these filamentous fungi produce various enzymes during growth (8–10), *qu* serves as an exogenous enzyme source during the production of fermented foods. Enzymes purified from *Aspergillus* sp. are also applied for medical industries. α-Amylase and lipase produced by *Aspergillus oryzae* have been used as digestive agents to treat dyspepsia (11). Furthermore, *Aspergillus* sp. produces ferulic acid esterase and releases free ferulic acid from the cell wall of plants (12, 13). Ferulic acid has been reported to show anti-inflammatory effects and accelerate gastrointestinal motility (14, 15). It has also been reported that free ferulic acid increased by fermentation with *qu* (16). Thus, the fermentation process by those filamentous fungi would add the stomachic function to the unfermented materials.

Chen et al. (17) has reported that *Aspergillus* sp. and *Rhizopus* sp. also exist in Chinese *shinkiku* products. Our previous study showed that *shinkiku* obtained from local markets in China and Korea contained digestive enzymes and ferulic acid (5). Therefore, digestive enzymes and ferulic acid derived by filamentous fungi possibly contribute to the stomachic property of *shinkiku*. However, in the previous study, neither *Aspergillus* sp. nor *Rhizopus* sp. could be detected using denaturing gradient gel electrophoresis (DGGE). It seems that DGGE is not appropriate for investigating the microbial structures of *shinkiku*. In addition, it was possible that *shinkiku* was stored in unsuitable environments in local markets, which altered the microbial conditions during storage. As *shinkiku* is prepared by microbial fermentation, characterization of microorganisms is required for quality control. In the present study, we obtained new commercial *shinkiku* products from a Japanese trading company specializing in crude drugs and investigated their chemical constituents and microbial structures using amplicon sequencing to establish reference standards for quality control of *shinkiku* in Japan.

## Materials and Methods

### Chemicals and Strains

Chemicals used for the analysis were obtained from Nacalai Tesque Inc. (Kyoto, Japan) and Wako Pure Industries Ltd. (Osaka, Japan). Folin and Ciocalteu's phenol reagent were purchased from MP Biomedicals (CA, USA), and ethylenediaminetetraacetic acid (EDTA) was obtained from DOJINDO (Kumamoto, Japan).

### Crude Drug Materials

**Figure 1:**
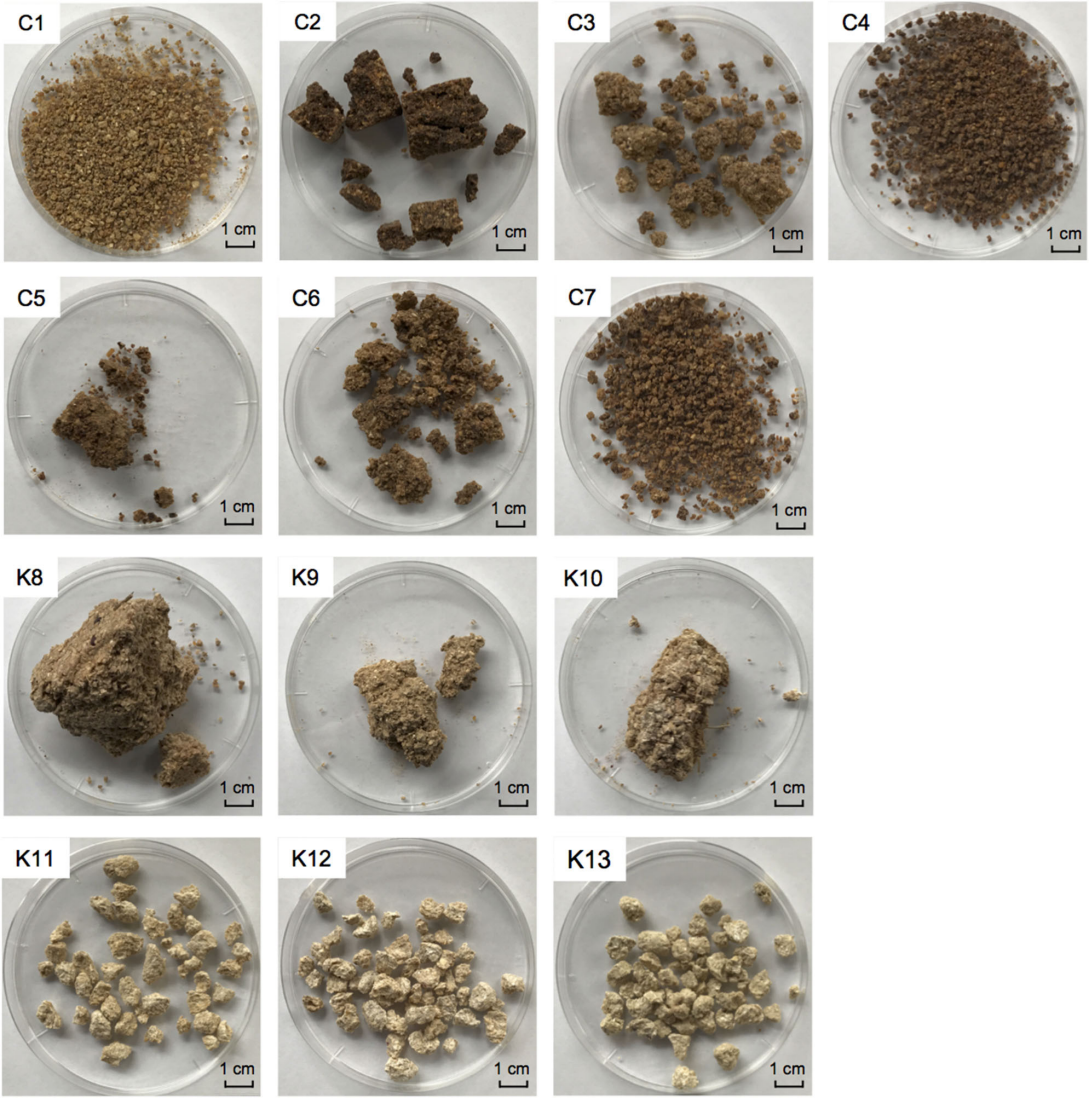
Commercial *shinkiku* products used in this study. Chinese products for C1–C7, and Korean products for K8–K13.

Thirteen commercial *shinkiku* products, imported from Korea or China, were kindly provided by Tochimoto Tenkaido Co., Ltd. (Osaka, Japan), Kotaro pharmaceutical Co., Ltd. (Osaka, Japan), and UCHIDAWAKANYAKU Ltd. (Tokyo, Japan). *Shinkiku* products used in this study are shown in Figure 1, and their production country and year of import are shown in Table 1. All the samples were maintained at −20°C until analysis.

**Table 1:** Commercial *shinkiku* products used in this study.

**Sample no**.	**Production area**	**Import year**
C1	China	2016
C2	China	2015
C3	China	2013
C4	China	2013
C5	China	2012
C6	China	2009
C7	China	2008
K8	Korea	2000
K9	Korea	1998
K10	Korea	1993
K11	Korea	2013
K12	Korea	2015
K13	Korea	2017

### DNA Extraction

DNA extraction was performed using a ZR Fecal DNA Miniprep kit (Zymo Research, CA, USA) according to the manufacturer's instructions. Mechanical disruption was carried out for each 100 mg of *shinkiku* at 6.0 m/s for 30 s by bead beating using a FastPrep 120 Cell Disrupter System (Thermo Savant; Carlsbad, CA, USA) and subjected to DNA extraction. The extracted DNA was quantified using a NanoDrop-8000 Spectrophotometer (Thermo Fisher Scientific K.K., Japan) and stored at −20°C until amplicon sequencing.

### Amplicon Sequencing Analysis

Amplicon sequencing analysis was performed at Bioengineering Lab. Co., Ltd. (Kanagawa, Japan). PCR amplification was performed using primer sets V3V4f and V3V4r for bacterial 16S rDNA V3/V4 regions and ITS1F_KYO1 and ITS2_KYO2 for the fungal ITS1 region (18, 19). For each sample, a library was prepared using the two-step tailed PCR method. The obtained amplicons were subjected to a 2 × 300 bp paired end run using a MiSeq system (Illumina K.K., Tokyo, Japan). The data were deposited to the DNA data Bank of Japan (DDBJ) under the accession numbers DRR205793-DRR205818. After quality filtering and chimera check of sequencing reads, the operational taxonomic units (OTU) were predicted using QIIME (Quantitative Insight Into Microbial Ecology) (20). The OTUs that accounted for more than 1% of the total sequence number were classified, whereas taxa with abundance <1% were summarized as “others.”

### Enzymatic Activity Assays

#### α-Amylase Assay

Crude enzyme extract was prepared according to the Official Analysis Method of National Tax Agency of Japan. *Shinkiku* (2 g) was suspended in 10 mL of 0.1 M acetate buffer solution (pH 5.0) containing 0.5% NaCl and homogenized at 11,000 rpm for 2 min on ice. Then, the homogenate was centrifuged at 22,300 × g at 4°C for 3 min. α-Amylase activity was measured using an α-amylase assay kit (Kikkoman Biochemifa, Tokyo, Japan) following the manufacturer's instructions, and 2-chloro-4-nitrophenyl-6^5^-azido-6^5^-deoxy-β-maltopentaoside (N3-G5-β-CNP) was used as a substrate, which was cleaved to 2-chloro-4-nitrophenol (CNP) on action of α-amylase. The increment of CNP was measured at 400 nm using a spectrophotometer. One unit of α-amylase activity was referred to the quantity of α-amylase required to release 1 μmol of CNP from N3-G5-β-CNP per minute at 37°C.

#### Protease Assay

Crude enzyme extract (10 mL) was dialyzed in a cellulose tube (Dialysis membrane 27/32, Viskase Companies Inc., USA) for 15 h in 1 L of 10 mM of acetate buffer (pH 5.0) and then filled up to 20 mL with water for the protease activity assay. A casein solution (2%, w/v) was used as a substrate, and the derived tyrosin by protease was quantified by a chromogenic reaction which used Folin and Ciocalteu's phenol reagent. Tyrosine was quantified using the standard curve obtained from absorbance of the known concentration of tyrosine standard. One unit of protease activity was defined as the quantity of protease required to release 1 μg of tyrosine from casein per hour at 40°C.

#### Lipase Assay

Lipase solution from *shinkiku* was prepared according to the method of Rahayu et al. (21) with slight modification. *Shinkiku* (5 g) was added to 15 mL of 0.1 M acetate buffer solution (pH 5.0) containing 0.5% NaCl and homogenized at 11,000 rpm for 1 min, and then, 10 mL of ethanol (99.5%) was added to the mixture and held for 1 h to extract. The supernatant obtained by centrifugation at 890 × g for 5 min was used as a lipase solution. The lipase activity was determined by Lipase Activity Assay Kit (Cayman Chemical, MI, USA) following the assay protocol. An arachidonoyl-1-thioglycerol was used as substrate, and thioglycerol derived by lipase was quantified using a fluorescence detector (ex. 380 nm, em. 510 nm) (Mithras LB940, Berthold, GmbH & co. KG., Germany) after reaction with the thiol detector. The thioglycerol concentration was determined using the calibration curve obtained from the fluorescence intensity of the known concentration of thioglycerol standard. One unit was defined as the amount of lipase that was required to release 1 nmol of thioglycerol from arachidonoyl-1-thioglycerol per minute at 37°C.

#### Quantification of Ferulic Acid

Ferulic acid (4-hydroxy-methoxycinnamic acid) was quantified according to the method of Okutsu et al. (5) with slight modifications. *Shinkiku* (2 g) was added to 10 mL of a water/methanol mixture (3:2) and was homogenized at 11,000 rpm for 1 min. The mixture was then centrifuged at 4,800 × g for 5 min. The supernatant was filtered through a 0.45-μm cellulose acetate membrane filter (Toyo Roshi Kaisha Ltd., Tokyo, Japan), and 10 μL of the sample was injected into the High Performance Liquid Chromatography (HPLC) system (Shimadzu LC system 20A, Shimadzu Corp., Kyoto, Japan). The ferulic acid concentration was determined using the calibration curve obtained from the peak area of the known concentration of ferulic acid standard.

### Quantification of Other Organic Acids

Organic acids except for ferulic acid were determined using a Prominence HPLC system and an electroconductivity detection (Shimadzu CDD-10AVP). Each *shinkiku* (2 g) sample was mixed with 20 mL of deionized water, homogenized at 11,000 rpm for 1 min, and then, centrifuged at 4,800 × g for 5 min to obtain a supernatant. The supernatant was filtered through a 0.45 μm pore-size cellulose acetate membrane filter. The separation of organic acids was performed using an ion-exclusion chromatography column (Shim-pack SCR-102H, 8 mm I.D. × 300 mm × 2) equipped with a Guard column (SCR-102H, 6 mm I.D. × 50 mm) at 50°C using 4 mM *p*-toluenesulfonic acid as the mobile phase at a flow rate of 0.8 mL/min. The buffer solution containing 16 mM Bis-Tris, 4 mM *p*-toluenesulfonic acid, and 80 μM EDTA was pumped into the column at 0.8 mL/min flow rate.

### Volatile Compounds Analysis

The supernatant used for organic acid analysis was also used to analyze the volatile compounds in *shinkiku* products. The volatile compounds were evaluated with the method described by Rahayu et al. (21). In brief, 10 mL supernatant, described in the quantification of organic acid, was transferred to a 10 mL sample vial with a 15-mm stir bar coated with 0.5 mm polydimethylsiloxane (Twister, GERSTEL K.K., Japan). The sample was stirred using a stir bar at 1,200 rpm for 60 min at 25–30°C to adsorb volatile components. Then, the stir bar was removed from the sample, washed with deionized water, and placed into a glass insert. The volatile compounds were desorbed from the SBSE stir bar using the temperature program of thermal desorption system (GERSTEL TDS 3, GERSTEL CIS 4, GERSTEL K.K., Japan) and transferred to a gas chromatograph (6890N Network GC System) equipped with a mass spectrometer (5975B insert MSD) (Agilent Technologies, Palo Alto, CA). The volatile compounds were separated on a Pure-WAX column (0.25 mm I.D. × 60 m length × 0.25 μm film thickness, J&W Scientific, Folsom, CA), and the oven temperature was held at 50°C for 5 min at 3°C/min till the temperature reached 240°C (hold for 5 min). The carrier gas was helium at a flow rate of 2 mL/min. The detected compounds were identified by comparison of their mass spectra with the National Institute of Standards and Technology (Gaithersburg, MD, USA) mass spectral library (NIST 05) and by comparison of their retention index (RI) with the database of Aroma Office software (Nishikawa Keisoku/Gerstel K.K., Japan).

## Results

### Microbial Structure in Shinkiku Products

Preliminary, we investigated the viable fungi in *shinkiku* products using a plating assay to detect *Aspergillus* sp., *Rhizopus* sp., or *Mucor* sp.; however, no filamentous fungus like colonies were detected except for C1, K11, and K13 (Table S1). Thus, amplicon sequencing analysis was applied to investigate the community structure of microorganism that included filamentous fungi in *shinkiku* products.

**Figure 2:**
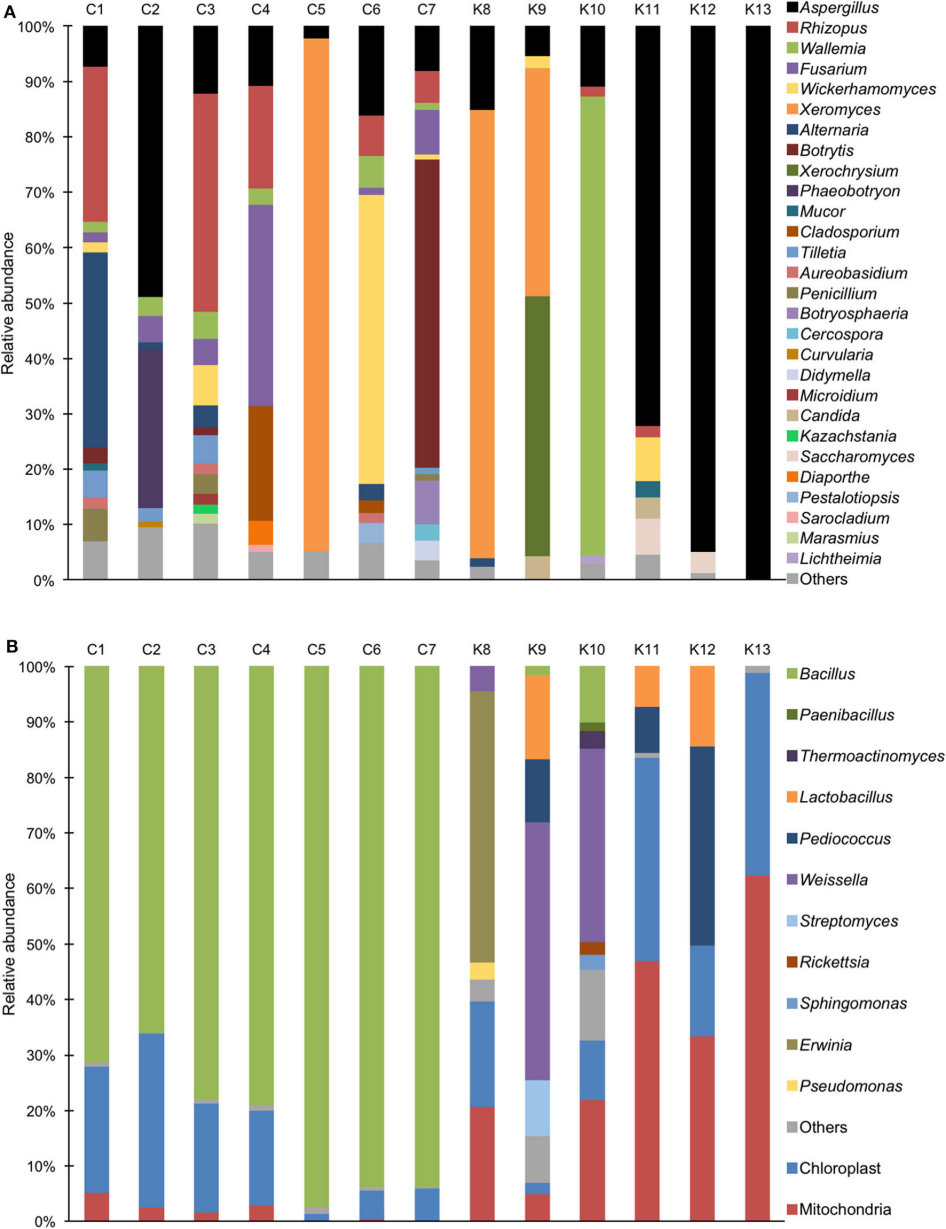
Relative abundance of bacterial and fungal sequence reads at the genus level in *shinkiku* products. **(A)** Fungi detected by ITS1F_KYO1-ITS2_KYO2 primer set; **(B)** Bacteria detected by V3/V4f-V3/V4r primer set.

*Aspergillus* sp. was detected in all *shinkiku* products, and *Rhizopus* sp. was found in more than half of *shinkiku* products (7/13) (Figure 2A). *Aspergillus* sp. and *Rhizopus* sp. were totally accounted for more than 50% in C3, K11, K12, and K13. *Rhizopus* sp. was predominant in C1, C3, and C4 compared with *Aspergillus* sp., but *Aspergillus* sp. was predominant in C2, K11, K12, and K13. In particular, *Aspergillus* sp. accounted for more than 70% in K11, K12, and K13. Meanwhile, *Mucor* sp. was detected only in K11 at 3%, and *Saccharomyces* sp. was detected in K11 and K12 at 4–7%. *Xeromyces* sp. was detected in C5, K8, and K9, and accounted for more than 40%. *Wallemia* sp. was detected in K10 and accounted for more than 80%. In addition, *Fusarium* sp. was detected in Chinese products such as C1, C2, C3, C4, C6, and C7, and accounted for 36% in sample C4. *Botrytis* sp. was characteristically detected in C7 with an abundance rate of 55%. *Wickerhamomyces* sp. was detected in C1, C3, C6, C7, K9, and K11 and accounted for 52% in C6.

For bacterial community structure, *Bacillus* sp. accounted for more than 60% in all Chinese products, but chloroplast and mitochondria DNA accounted for more than 50% in Korean products such as K11, K12, and K13 (Figure 2B). Multiple lactic acid bacteria were detected in Korean products. *Pediococcus* sp. and *Lactobacillus* sp. were detected in K12, and *Weissella* sp. was detected in K9 and K10. In contrast, *Erwinia* sp. was detected in K8.

### Enzyme Activities in *Shinkiku* Products

As *shinkiku* is used to treat anorexia and dyspepsia, digestive enzymes derived by microorganisms in *shinkiku* work as a digestive promotor (22). Thus, we measured the digestive enzyme activities in *shinkiku* products. All *shinkiku* products showed α-amylase (8.3–51.3 U/g), protease (86.0–7892.8 U/g), and lipase (0.1–5805.5 U/g) activity, and Korean products tended to have higher enzyme activity than Chinese products (Table 2). K8 and K10 showed higher amylase activity compared to other *shinkiku* products. K13 showed the highest protease activity, and K11 showed the highest lipase activity among all *shinkiku* products.

**Table 2:** Digestive enzyme activities of *shinkiku* products.

**Sample**	**U/g**		
	**α-Amylase**	**Protease**	**Lipase**
C1	10.2	560.8	2.6
C2	10.3	86.0	0.1
C3	8.3	591.7	11.4
C4	35.4	435.9	1.9
C5	9.8	328.3	25.8
C6	10.0	116.1	5.5
C7	8.3	260.6	11.9
K8	51.3	2,029.8	1,673.8
K9	35.8	1,858.2	696.7
K10	49.6	2,814.6	2,656.5
K11	19.7	1,552.7	5,805.5
K12	11.8	1,762.6	4,025.0
K13	21.1	7,892.8	2,639.7

### Ferulic Acid in *Shinkiku* Products

Ferulic acid could contribute to efficacies of *shinkiku* because it accelerates gastrointestinal motility (15). Because some microorganisms secrete ferulic acid esterase (13, 23), free ferulic acid could be released from wheat materials during *shinkiku* fermentation. All *shinkiku* products were confirmed to contain free ferulic acid, and the content ranged from 25.8 to 91.4 nmol/g in Korean products and from 99.9 to 554.6 nmol/g in Chinese products (Table 3).

**Table 3:** Organic acid contents of *shinkiku* products.

**Sample**	**nmol/g**	**μmol/g**					
	**Ferulic acid**	**Citric acid**	**Pyruvic acid**	**Lactic acid**	**Formic acid**	**Acetic acid**	**Total**
C1	99.9	8.0	4.6	9.5	4.3	5.6	032.1
C2	321.0	7.8	8.4	80.3	7.0	7.7	111.2
C3	87.8	7.7	10.7	16.3	5.0	5.9	045.7
C4	554.6	8.6	10.5	38.3	6.9	8.2	072.6
C5	140.7	8.0	13.5	23.8	7.3	3.3	056.0
C6	109.8	9.7	5.4	11.4	6.8	6.9	040.2
C7	176.5	9.6	14.3	9.2	8.7	5.7	047.6
K8	60.7	8.1	6.0	0.7	n.d.	0.5	015.3
K9	42.3	3.7	0.2	2.3	n.d.	0.5	006.6
K10	91.4	3.8	n.d.	1.0	0.8	0.4	005.9
K11	75.4	72.6	n.d.	11.5	1.9	3.1	089.2
K12	59.9	88.8	n.d.	7.0	2.8	2.0	100.6
K13	25.8	71.9	n.d.	0.9	4.5	1.1	078.5

n.d., not detected.

### Other Organic Acids in *Shinkiku* Products

Organic acids are important metabolites of fermentative microorganisms. As each microorganism secretes different organic acids, we quantified the organic acids to investigate the relationship with microbial structure in *shinkiku* products. Citric acid, pyruvic acid, lactic acid, formic acid, and acetic acid were detected in *shinkiku* products (Table 3). Although K11, K12, and K13 contained remarkably high citric acid (71.9–88.8 μmol/g), the total organic acid contents tended to be higher in Chinese products compared to Korean products. Especially C2, C4, and C5 contained higher lactic acid compared to other *shinkiku* products.

### Volatiles in *Shinkiku* Products

**Figure 3:**
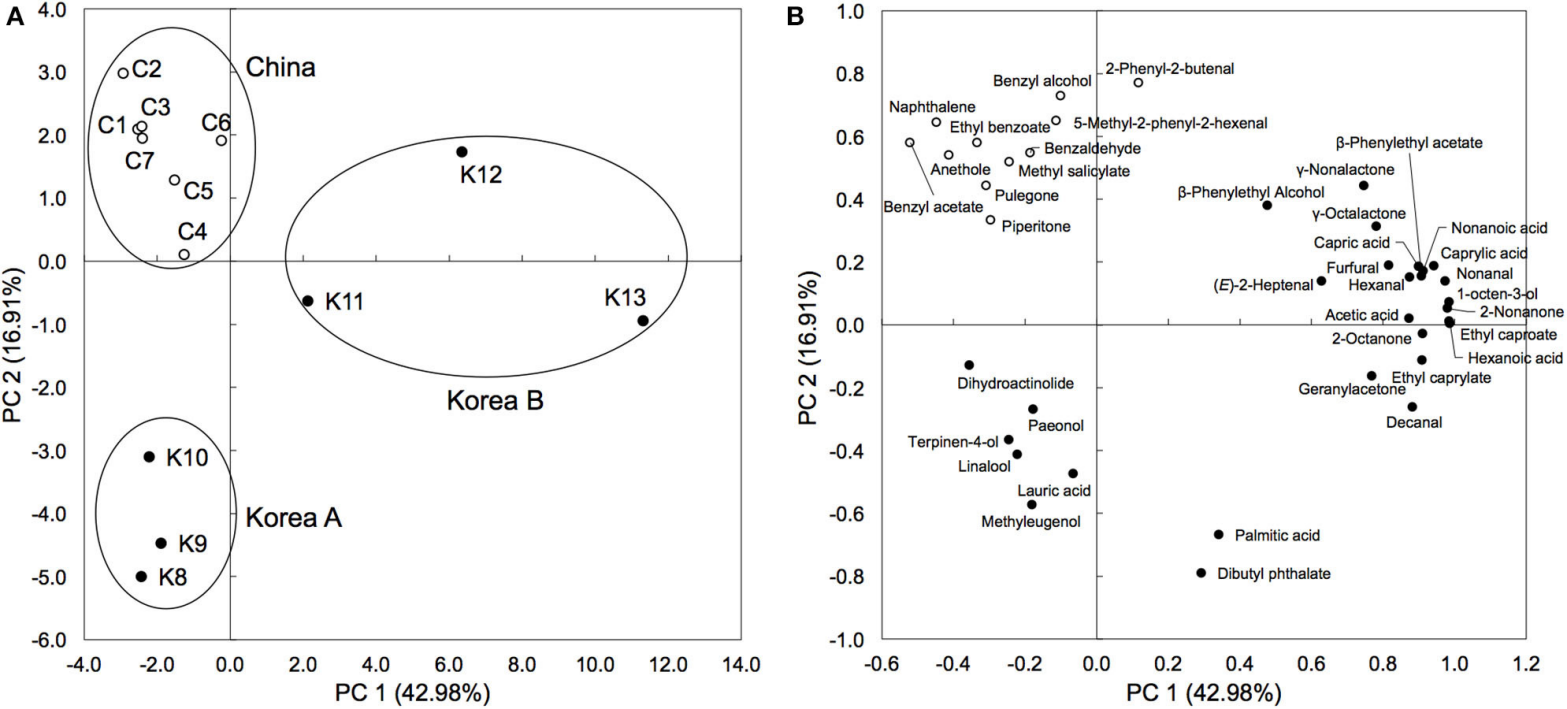
Principal component analysis based on peak area of common volatile components in *shinkiku* products. **(A)** is for Score plot, and **(B)** is for Loading plot.

Herb materials in *shinkiku* contain various essential oils, and they act as appetizers (stomachic) (24). We measured the volatile content in *shinkiku* products exhaustively using GC-MS. A total of 146 compounds were detected, and among them, 39 compounds were common in all Chinese and Korean products: 7 acids, 5 alcohols, 8 aldehydes, 7 esters, 5 ketones, 3 lactones, 3 phenols, and 1 polycyclic aromatic hydrocarbon (Table 4). Principal component analysis (PCA) were used to analyze datasets of peak area of 39 common volatiles to differentiate *shinkiku* products. The PCA score plot indicated a clear variance for different volatiles based on different manufacture across PC1 (42.98%) and PC2 (16.91%) (Figure 3).

**Table 4:** Volatile compounds commonly detected in shinkiku products.

**Name**	**RI^**a**^**	**m/z**	**Identification**	**Peak area (1/1.0 × 10**^**6**^**)**^b^		
				**minimum**	**maximum**	**mean^**c**^**
**Acids**						
Non-anoic acid	2,147	73	MS, RI	1.8	107.8	24.5
Capric acid	2,253	73	MS, RI	3.2	23.5	9.8
Lauric acid	2,464	73	MS, RI	8.7	22.9	12.3
Acetic acid	1,439	60	MS, RI	1.4	10.6	2.8
Hexanoic acid	1,829	60	MS, RI	1.0	407.7	70.3
Palmitic acid	2,881	73	MS, RI	131.0	479.5	268.3
Caprylic acid	2,041	60	MS, RI	1.4	105.3	24.6
**Alcohols**						
Linalool	1,538	93	MS, RI	3.3	48.0	15.9
Terpinen-4-ol	1,592	93	MS, RI	1.8	16.9	8.1
Benzyl alcohol	1,858	108	MS, RI	3.6	117.3	51.1
1-Octen-3-ol	1,442	57	MS, RI	5.3	158.7	33.7
β-Phenylethyl Alcohol	1,893	91	MS, RI	4.0	37.4	10.7
**Aldehydes**						
(*E*)-2-Heptenal	1,315	83	MS, RI	1.0	15.4	4.9
Benzaldehyde	1,509	106	MS, RI	11.1	4,610.7	1,013.1
2-Phenyl-2-butenal	1,912	146	MS, RI	0.8	13.2	6.1
5-Methyl-2-phenyl-2-hexenal	2,056	117	MS, RI	0.7	36.1	11.6
Hexanal	1,085	56	MS, RI	6.3	427.1	96.2
Nonanal	1,384	57	MS, RI	2.7	64.8	17.2
Decanal	1,489	57	MS, RI	2.4	18.9	7.4
Furfural	1,451	96	MS, RI	1.4	89.5	15.3
**Esters**						
Ethyl benzoate	1,655	105	MS, RI	1.0	60.8	13.9
Dibutyl phthalate	2,676	149	MS, RI	11.8	129.3	49.3
Benzyl acetate	1,715	108	MS, RI	1.0	46.7	19.5
Ethyl caproate	1,229	88	MS, RI	0.5	480.2	75.9
Ethyl caprylate	1,428	88	MS, RI	1.0	33.7	9.1
Methyl salicylate	1,760	120	MS, RI	1.9	22.1	7.4
2-Phenylethyl acetate	1,801	104	MS, RI	2.1	37.1	12.9
**Ketones**						
Pulegone	1,635	152	MS, RI	2.7	66.1	14.9
Piperitone	1,713	110	MS, RI	1.9	181.8	23.4
2-Octanone	1,279	58	MS, RI	1.6	71.0	11.1
2-Nonanone	1,381	58	MS, RI	0.8	30.2	5.7
Geranylacetone	1,842	69	MS, RI	3.9	14.9	7.0
**Lactones**						
γ-Octalactone	1,897	85	MS, RI	0.8	32.4	10.8
γ-Nonalactone	2,009	85	MS, RI	13.6	203.5	94.8
Dihydroactinolide	2,319	111	MS, RI	2.8	13.3	6.0
**Phenols**						
Anethole	1,811	148	MS, RI	3.8	100.5	28.5
Paeonol	2,237	151	MS, RI	35.6	5,626.8	807.8
Methyleugenol	1,994	178	MS, RI	3.6	5,499.0	452.4
PAH*						
Naphthalene	1,724	128	MS, RI	3.7	45.8	16.4

* *PAH, polycyclic aromatic hydrocarbon*.

a *RI, retention index calculated on a Pure-WAX column*.

b *Peak area was shown as a relative value with 1 × 10^6^*.

c *Mean value of component peak area was calculated on the average of peak areas in 13 shinkiku*.

A clustered pattern was observed in Chinese products with a higher content of some benzenoid aromatic hydrocarbons such as naphthalene, ethyl benzoate, benzaldehyde, and benzyl alcohol. The Korean products were further separated into two groups. Samples K8, K9, and K10 were classified into the same group (Korea A) because of the higher content of terpenoids such as linalool, paeonol, and terpinen-4-ol. Meanwhile, samples K11, K12, and K13 were differentiated (Korea B) from other groups with higher contents of fatty acid (hexanoic acid, capric acid, and caprylic acid), aldehydes (decanal and hexanal), and alcohols (β-phenylethyl alcohol and 1-octen-3-ol). In addition to the common volatiles, 2,3-butanediol and pyrazines were characteristically detected in all Chinese products. Vanillin and 4-vinylguaiacol, which are metabolites of ferulic acid, were also detected in all Chinese products. Meanwhile, *d*-limonene, myristic acid, toluene, 2-pentylfuran, 2-heptanone, hinesol, and methylguaiacol were only detected in all Korean products (data not shown).

## Discussion

Amplicon sequencing analysis revealed that *Aspergillus* sp. was common microorganism in all *shinkiku* products, and no common bacterial types was detected in all products. Thus, *shinkiku* was manufactured with microbial fermentation with such filamentous fungi. As these fungi were difficult to be detected as viable cells, they possibly died during the production or storage process of *shinkiku*. Thus, amplicon sequencing analysis seems appropriate for clarifying the microbial structure in *shinkiku*. In addition, digestive enzymes (α-amylase, protease, and lipase), organic acids (ferulic acid, citric acid, lactic acid, and acetic acid), and 39 volatiles were commonly found in *shinkiku* products. These microbial and chemical characteristics of *shinkiku* is fundamental data for the quality control of *shinkiku* in the market, and the commonalities would contribute to normalize the quality of *shinkiku*.

However, microbial and chemical characteristics of *shinkiku* were considerably different among the *shinkiku* products. Various fungi were detected in Chinese products. Not only *Aspergillus* sp. and *Rhizopus* sp. but also *Mucor* sp. and *Saccharomyces* sp. were detected with a small abundance. These microorganisms have been reported to exist in Chinese *qu* (25, 26). *Qu* is mixed as a fermentation starter for *shinkiku* manufacture in China. The microbial community of *qu* was reported to be different among the products because it was prepared by natural fermentation (8, 27). Thus, the microbial diversity of *qu* possibly affects the microbial structures of *shinkiku* in China.

For bacteria communities, *Bacillus* sp. was predominant in all Chinese products. As *Bacillus* sp. is generally found in soil and grass hay, it possibly comes from wheat bran or herb materials in *shinkiku*. *Bacillus* sp. is reported to secret considerable ferulic acid esterase (23). It is expected that the higher ferulic acid in Chinese products was caused by ferulic acid esterase secreted by *Bacillus* sp. Higher levels of benzenoid compounds such as benzaldehyde, and anethole in Chinese products were also derived by *Bacillus* sp. because these volatiles have been reported to be synthesized by *Bacillus* sp. (28, 29).

In the case of Korean products, *Aspergillus* sp. was more predominant than *Rhizopus* sp., and microbial composition was simpler compared to Chinese products. It suggests that pure cultivated *Aspergillus* sp. might be inoculated in the materials to ferment *shinkiku* in Korea. In addition, a relatively high abundance of chloroplast DNA from herb materials and that from mitochondria were detected in Korean products. This result suggests that the amount of DNA derived from bacteria was extremely low in Korean products. The number of viable bacteria in Korean products was also considerably lower than that in Chinese products (Table S1). It was reported that the number of viable bacteria in *qu* was increased at the late stage of fermentation (30). Therefore, it was expected that Korean *shinkiku* was short-fermented with simple microorganisms.

Lactic acid bacteria such as *Weissella* sp., *Lactobacillus* sp., and *Pediococcus* were detected in Korean products. As these lactic acid bacteria are known to have potential to promote digestion for humans (31, 32), they possibly contribute to the efficacies of Korean *shinkiku* for anorexia and dyspepsia. However, the relative abundances of lactic acid bacteria were inconsistent with lactic acid contents in *shinkiku*. In addition, *Rhizopus* sp. in *qu* was also reported to secrete lactic acid (33), but the relative abundance of *Rhizopus* sp. was inconsistent with lactic acid content in *shinkiku* products. It possible that the microbial structure changed during fermentation, and the microorganisms in the early stage of the fermentation process affect the lactic acid contents in *shinkiku* products. Although our study revealed the microbial structure only in the final product of *shinkiku*, further studies are needed to investigate the changes in microbes and their metabolites during *shinkiku* fermentation.

PCA based on the volatile components differentiated *shinkiku* products into three groups, among them two groups were composed of Korean *shinkiku* (Korea A and Korea B). The relative abundance of *Aspergillus* sp. in Korea B was considerably higher than that in Korea A. High levels of fatty acids in Korea B were possibly caused by lipase because of their higher lipase activity. The higher citric acid content in Korea B was also caused by its high abundance of *Aspergillus* sp. because *Aspergillus* sp. is known to secrete considerable digestive enzymes and citric acid (34). Therefore, the microbial characteristics were found to affect the chemical composition of *shinkiku*.

In contrast, some contaminating microorganisms were also detected in several *shinkiku* products. *Xeromyces* sp. and *Wallemia* sp. are known as food-contaminating fungi, and they were reported to have drought-resistance (35, 36). *Fusarium* sp. is a type of plant pathogenic fungi that produces mycotoxin (37). *Erwinia* sp. is known as a pathogenic bacteria of plants (38), and thus, *Erwinia* sp. detected in *shinkiku* products was possibly derived from infected herb materials. This result suggests that some samples in the market were possibly contaminated with poisonous microorganisms, and thus, microbial control would be critically important in *shinkiku* production.

In addition to the microbial structure, manufacturing conditions seemed to be different among manufacturers. *Bacillus* sp., which is predominant in Chinese products, forms heat-resistant spores (39), and thermal treatment caused the selective survival of *Bacillus* sp. (40). The lower enzyme activities in Chinese products indicated the possibility of enzyme deactivation by heat treatment. In addition, pyrazines, which are produced during heating (41), were also detected in Chinese products. Thus, Chinese products should be thermally treated for dryness after fermentation. In contrast, heat treatment should not be carried out for Korean products because enzyme activities in Korean products are higher than those in Chinese products. Korean products, especially Korea A, contained higher terpenoids such as linalool and terpinen-4-ol. They were derived from essential oils in herbal materials in *shinkiku*: wormwood (42), cocklebur (43), and polygonum (44). As these terpenoids are volatile, Korean *shinkiku* is possibly dried at lower temperature than Chinese *shinkiku*. In addition, ferulic acid content was relatively lower in Korean products in spite of the higher abundance of *Aspergillus* sp., which is also reported to secret ferulic acid esterase (12, 13). As ferulic acid is an enzymatic product, the production temperature and duration possibly affected the ferulic acid content in *shinkiku*. Further studies are needed to investigate the relationships between manufacturing processes and chemical constituents in *shinkiku*.

Although there was no relationship between the import year and quality of *shinkiku* (Table 1), some *shinkiku* products contained vanillin, which is possibly formed during storage. Vanillin is derived from 4-vinylguaiacol by the maturation of liquors such as *awamori* (45). As vanillin formation is affected by temperature, the storage condition of *shinkiku* would affect the vanillin content. The effects of storage and transportation were also confirmed by comparing the effects observed in the previous study. Digestive enzyme activities and ferulic acid contents of some *shinkiku* obtained from local markets were significantly lower than those in the present study (5). As *shinkiku* seems to be stored for a long time in unsuitable environment in the local markets, the enzyme activities or ferulic acid content were possibly altered depending on the storage condition. Further studies are required to investigate the aging effects on the quality of *shinkiku*.

In conclusion, our study revealed that the commonality and diversity of commercial *shinkiku* products. The commonalities were possibly the reference standard for quality control of *shinkiku*. Furthermore, amplicon sequence analysis showed a clearly different microbial characteristic among the products. As a result of chemical analysis partially corresponding to microbial structure, the microorganisms in *shinkiku* were found to affect its chemical composition. Thus, microbial management was suggested to be important to stabilize the quality of *shinkiku* products. In addition, the results indicated that manufacturing conditions such as heating temperature seemed to be different among manufacturers. To standardize the *shinkiku* quality, further studies are needed to elucidate the effects of microbes or manufacturing conditions on chemical constituents of *shinkiku*.

## Data Availability Statement

The datasets generated for this study can be found in the DNA data Bank of Japan (DDBJ)/DRR205793-DRR205818.

## Author Contributions

ZW and KO performed all chemical analyses and wrote the original draft. YY, KK, FH, and KTa contributed to the conception and design of the experiment and paper preparation. TF and HT supervised microbial analysis and reviewed the drafts of the paper. TM and KTo performed sample collection and reviewed drafts of the paper. All authors contributed to the article and approved the submitted version.

## Conflict of Interest

The authors declare that the research was conducted in the absence of any commercial or financial relationships that could be construed as a potential conflict of interest.
