# Supplementary material for: Microbial Community Structure and Chemical Constituents in Shinkiku, a Fermented Crude Drug Used in Kampo Medicine
Source: Front Nutr. 2020 Jul 31;7:115. doi: 10.3389/fnut.2020.00115 (PMC7416650; doi:10.3389/fnut.2020.00115)
Supplement: Supplementary file 1 [file Table_1.DOCX]

Supplementary Material

## Supplementary Table

**Table S1.** Total viable cell count in *shinkiku* products.

|  | | | |  |
| --- | --- | --- | --- | --- |
| Sample | Total viable cell count (cells/g) | |  |  |
|  | Filamentous fungi^a^ | Viable bacteria^b^ |  |  |
| C1 | 1.0×10^2^ | 1.3×10^8^ |  |  |
| C2 | <10^2^ | 1.2×10^8^ |  |  |
| C3 | <10^2^ | 1.1×10^8^ |  |  |
| C4 | <10^2^ | 7.8×10^8^ |  |  |
| C5 | <10^2^ | 1.0×10^8^ |  |  |
| C6 | <10^2^ | 1.1×10^8^ |  |  |
| C7 | <10^2^ | 1.0×10^8^ |  |  |
| K8 | <10^2^ | 2.5×10^3^ |  |  |
| K9 | <10^2^ | 7.0×10^2^ |  |  |
| K10 | <10^2^ | 3.1×10^3^ |  |  |
| K11 | 3.5×10^2^ | 5.8×10^3^ |  |  |
| K12 | <10^2^ | 4.7×10^3^ |  |  |
| K13 | 1.5×10^6^ | 2.5×10^2^ |  |  |

^a^: Appropriately diluted suspension of *shinkiku* was plated on to *Koji*-extract medium (Ishiyama et al., 2008) contains 0.1% triton, and the number of colonies were counted after incubation at 30°C for 42-46 h.

^b^: Appropriately diluted suspension of *shinkiku* was plated on to Anti-Fungus Culture Medium (DAIGO, Wako Pure Industries Ltd., Osaka, Japan), and the number of colonies were counted after incubation at 30°C for 42-46 h.
